# Intraductal tubular papillary neoplasm (ITPN), a novel entity of pancreatic epithelial neoplasms and precursor of cancer: A case report and review of the literature

**DOI:** 10.1016/j.ijscr.2019.01.036

**Published:** 2019-02-05

**Authors:** Stefan Fritz, Regina Küper-Steffen, Katharina Feilhauer, Christof M. Sommer, Götz M. Richter, Alexander Bosse, René Hennig, Jörg Köninger

**Affiliations:** Department of General, Visceral, Thoracic and Transplantation Surgery, Department of Pathology, Clinics for Diagnostic and Interventional Radiology, Katharinenhospital Klinikum Stuttgart, Germany

**Keywords:** Intraductal tubular papillary neoplasm, ITPN, Pancreas, Cancer risk, Cystic tumors of the pancreas

## Abstract

**Background:**

Intraductal tubular papillary neoplasm (ITPN) displays a very rare subtype of epithelial neoplasms of the pancreas. ITPN is characterized by intraductal tubulopapillary growth and cellular dysplasia. In contrast to intraductal papillary neoplasm (IPMN) no overt epithelial mucin production is observed. To date, little is known about ITPN and particularly about pancreatic cancer arising in this tumor entity.

**Case presentation:**

A 68-year-old male presented at our hospital with a distal bile duct occlusion suspicious for adenocarcinoma of the pancreatic head. Preoperative staging revealed no signs of distant metastasis. The patient was surgically explored and pylorus preserving duodenopancreatectomy was performed for a solid pancreatic head tumor. Final histopathology surprisingly revealed an ITPN with an associated invasive carcinoma pT3, pN0 (0/12), R0, G2.

**Discussion:**

Patients with ITPN frequently present with jaundice suspicious for a bile duct stenosis or a malignant tumor of the pancreatic head. Although, it is possible to diagnose ITPN by endoscopic retrograde cholangiopancreaticography, many tumors are found not before histopathological examination. Differential diagnosis includes ductal adenocarcinoma of the pancreas, neuroendocrine tumors, IPMN, distal bile duct tumors, and solid pseudopapillary neoplasms. Using immunohistochemistry, other entities of pancreatic tumors can be ruled out. In case of R0 resection oncological prognosis is described to be more favorable when compared to regular ductal adenocarcinoma.

**Conclusion:**

ITPN displays a rare entity of pancreatic neoplasms. As shown in the present case report, there is a relevant potential of malignant transformation and therefore radical surgical resection and oncologic follow-up is warranted.

## Highlights

• Intraductal tubular papillary neoplasm (ITPN) displays a very rare subtype of epithelial neoplasms of the pancreas.
• To date, little is known about ITPN and particularly about pancreatic cancer arising in this tumor entity.
• ITPN reveals a potential of malignant transformation. Radical surgical resection and oncologic follow-up is warranted.
• When compared to ductal adenocarcinoma, patients with ITPN reveal less lymph node metastasis and a better overall survival.

## 1 Background

Intraductal tubular papillary neoplasm (ITPN) displays a rare subtype of epithelial neoplasms of the pancreas. This entity has been described for the first time in 2009 by Yamaguchi et al. [1]. In the most recent 4^th^ edition of WHO classification 2010, ITPNs were recognized as a distinct entity of pancreatic tumors. They were included into the subgroup of premalignant epithelial tumors. Two different types of intraductal neoplasms of the pancreas were noted in the 2010 WHO classification: intraductal tubular papillary neoplasm (ITPN) and intraductal papillary mucinous neoplasm (IPMN) [2].

While IPMN has become a clinically well described and established entity of cystic pancreatic neoplasms [3,4], very little is known about ITPN and particularly about pancreatic cancer arising in ITPN [5]. According to the 2010 WHO classification, ITPN are intraductal, grossly visible, tubule-forming epithelial neoplasm with high-grade dysplasia and ductal differentiation. In contrast to IPMNs, no overt production of mucus is observed in ITPN [2]. In case the tumor displays invasive tumor growth, it is referred to as an “ITPN with an associated invasive carcinoma” [2].

Due to its rarity, data about the natural history of ITPN and particularly about pancreatic cancer arising in ITPN is very limited [5]. Moreover, there are only sporadic reports about prognosis and oncologic outcome following resection of malignant ITPN [6]. This is a case report of a 68-year-old male suffering from adenocarcinoma of the pancreas arising in ITPN with review of the current literature focusing on the malignant potential of this tumor entity. Written informed consent was obtained from the patient for publication of this case report and accompanying images. A copy of the written consent is available for review by the Editor-in-Chief of this journal on request. The present case has been reported in accordance with the SCARE criteria [7].

## 2 Case presentation

**Fig. 1:**
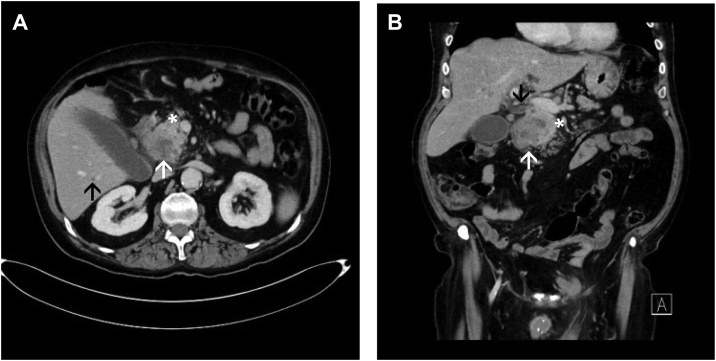
Preoperative computed tomography (CT). Inhomogeneous hypodense ill-defined focal lesion of the head of the pancreas (white arrows) with a dilated intra- and extrahepatic bile duct system due to a mass effect (black arrows) (A transverse image, porto-venous phase; B coronal image, porto-venous phase). Note the healthy adjacent parenchyma of the pancreas (white asterisks) and lack of signs of local irresectability.

**Fig. 2:**
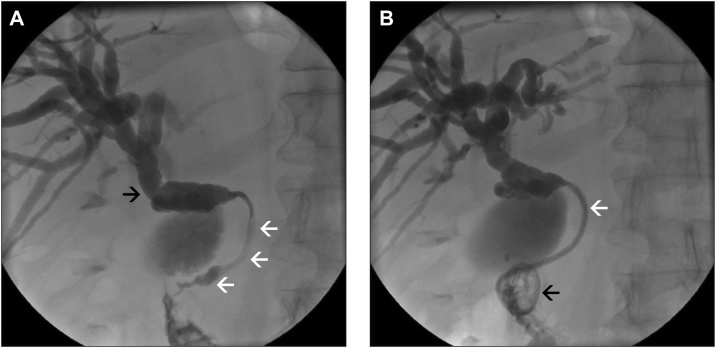
Percutaneous transhepatic cholangiodrainage (PTCD). Conventional percutaneous cholangiogram with opacification of a dilated intra- and extrahepatic bile duct system (black arrow) with signs of a malignant stenosis at the level of the head of the pancreas (white arrows) (A). Control cholangiogram after positioning of a 8 F PTCD for combined external/internal drainage (black arrow: configuration of the pigtail of the PTCD in the duodenum; white arrows: bridging of the malignant obstruction via the 8 F PTCD) (B).

A 68-year-old male presented at an external hospital with painless obstructive jaundice and a loss of weight of 5 kg over the last two months. Except for a Billroth II gastric resection in 2011 for ulcer disease, no previous abdominal surgery was noted. Secondary diagnoses included a smoking history of 10 pack years, arterial hypertension, and a mild type 2 diabetes treated with metformin. The diabetes lasted for several years and was not new onset. Using contrast-enhanced multi-phase computed tomography (CT), a tumor in the head of the pancreas associated with a distal biliary obstruction was diagnosed suspicious for pancreatic cancer (Fig. 1). The Ca 19-9 serum level was extensively elevated with 2100 U/ml. Since there were no imaging signs of distant metastasis nor signs of local irresectability, the patient underwent surgical exploration on September 18, 2017 at the external hospital. Due to questionable infiltration of the caval vein and suspicious lymphadenopathy, the exploration was broken off. Histology of a sample biopsy of the hepatoduodenal ligament revealed fragments of a moderately differentiated adenocarcinoma. Postoperatively, due to persistent jaundice, the patient received percutaneous transhepatic bile duct drainage (PTCD) for combined external/internal drainage which was changed on October 6, 2017 to a self-expanding metal stent (SEMS) for permanent recanalization of the bile duct (Fig. 2). With regard to the suspected locally advanced ductal adenocarcinoma of the pancreas, a palliative systemic chemotherapy was anticipated.

**Fig. 3:**
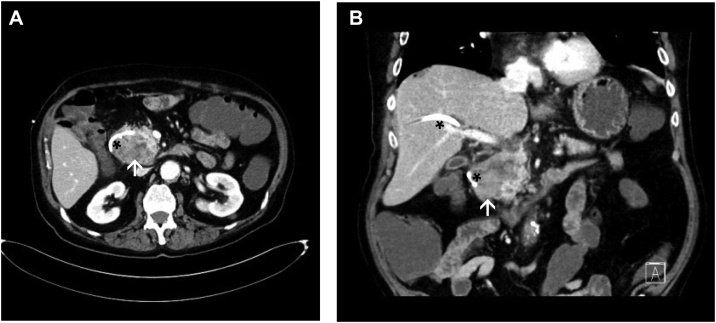
Computed tomography following PTCD. Control CT with adequate positioning of the PTCD (black asterisks) and relief of the bile duct system, and constant ill-defined tumor in the head of the pancreas (white arrows) (A transverse image, arterial phase; B coronal image, porto-venous phase).

Subsequently, the patient presented at the Katharinenhospital Stuttgart as a referral center for hepatobiliary and pancreatic surgery seeking a second medical opinion. The patient again underwent staging, which revealed no signs of liver or lung metastasis (Fig. 3). Upon an interdisciplinary tumor board decision, the patient was surgically re-explored on October 24, 2017. Intraoperatively, no contraindications against pancreatic head resection, and particularly no infiltration of the caval or portal vein were found. Thus, the patient underwent pylorus-preserving duodenopancreatectomy. The early post-operative course was uneventful. However, on post-operative day 10, the patient had to be reexplored due to an ischemic perforation of the transverse colon. A discontinuity resection of the transverse colon was performed with preparation of a colostomy and a long Hartmann stump. The further clinical course was uncomplicated and the patient was discharged on postoperative day 30 in good health condition.

**Fig. 4:**
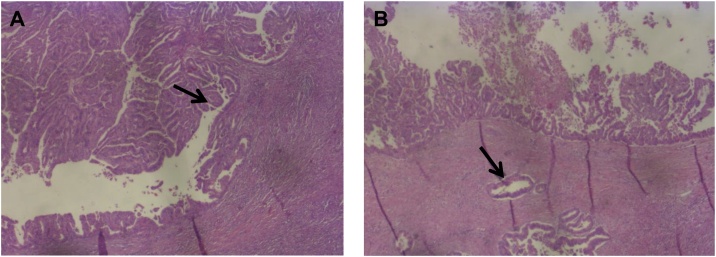
Histopathology (hematoxylin-eosin staining). (A) Histopathology of the pancreatic head following Whipple´s procedure reveals an intraductal tubulopapillary neoplasm (ITPN) with typical papillary growth and beginning invasion (arrow). In contrast to IPMN no overt mucin production was observed. (Original magnification, x 25). (B) Intraductal tubulopapillary neoplasm (ITPN) with associated invasive ductal adenocarcinoma (arrow). (Original magnification, x 25).

**Fig. 5:**
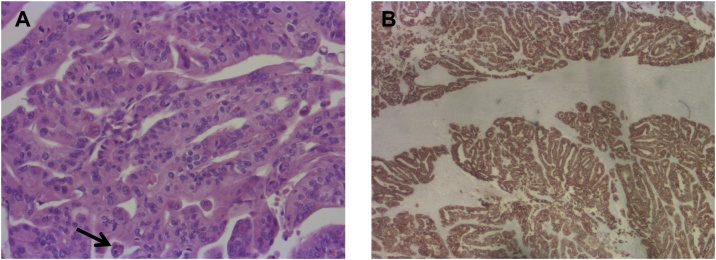
Hematoxylin-eosin staining and immunohistochemistry with Cytokeratin 7. (A) Hematoxylin-eosin staining in a higher magnification reveals atypical tumors cells with high-grade dysplasia and a high proliferation rate (arrow). (Original magnification, x 100). (B) Immunohistochemical staining of the tumor shows ubiquitously positive results for Cytokeratin 7 (CK7) as marked in brown color indicating a highly malignant behavior of the tumor. (Original magnification, x 25).

Final histopathology of the surgical specimen surprisingly revealed a pT3, pN0 (0/12), R0, G2 ductal adenocarcinoma arising in the background of an ITPN (Fig. 4). The tumor had a maximum diameter of 5.9 cm and showed wide infiltration of the duodenum. All resection margins were clear and lymph nodes were free of metastasis. To rule out a neuroendocrine tumor, immunohistochemistry was performed using Cytoceratin-7, Chromogranin, Synaptophysin, and Trypsin (Fig. 5). Since the tumor did not show expression of these markers, a neuroendocrine tumor and an acinus cell differentiation were excluded. According to the postoperative tumor board decision, adjuvant systemic chemotherapy with Gemcitabine and Xeloda was administered. The therapy was well tolerated by the patient and no severe complications were observed.

A follow-up re-staging CT in March 2018 revealed no signs of recurrent disease or distant metastasis. The patient therefore underwent restoration of intestinal continuity by an ascendodescendostomy. The postoperative course was uneventful and the patient was discharged on postoperative day six. To date the patient is in a good clinical condition without signs of recurrent disease and with a non-insulin dependent diabetes mellitus treated orally with metformin as preexisting prior to surgery.

## 3 Discussion

Clinical data about ITPN remains very limited. ITPN is a rare entity and was not recognized in literature before 2009. It is estimated that ITPN accounts for less than 1% of all pancreatic exocrine neoplasms and only approximately 3% of intraductal neoplasms [1,8]. In 2016, Date et al. conducted a literature research and described clinicopathological features of 58 cases of ITPN in the pancreas. Patients´ age ranged between 35 and 84 years with a mean of 61 years. In 59% of the cases, the tumor was located in either the head of the pancreas alone or in the head and body. In 33% the tumor was found in the body and/or tail of the pancreas, and in three cases (5%) the tumor affected the whole pancreas [9]. In the series of Kolby et al. in 14% of ITPN cases the entire pancreatic gland was involved [6]. Reported clinical symptoms included abdominal pain, jaundice, exacerbation of diabetes mellitus, and loss of appetite and weight [9]. Umemura et al. described a rare case of an ITPN rupturing and causing acute peritonitis [10]. As far as it is known, there is no sex predilection and ITPNs are not associated with any specific clinical syndrome [6[9]. In case of early manifestations of ITPN, clinical symptoms are frequently unspecific including general discomfort or mild abdominal pain. In these cases, the lesion is often detected more or less incidentally on abdominal imaging [11].

ITPN is frequently compared with IPMN [1,4]. Both entities display pancreatic neoplasms with intraductal growth and a relevant potential for malignant transformation. However, there are important differences that can be used to reliably distinguish between these two tumor entities. First of all, IPMNs typically reveal excessive mucin production whereas ITPN does not show this attribute. Second, ITPN is characterized by a distinct tubule-forming epithelium that can be observed on routine hematoxylin eosin staining and is exclusively seen in ITPN [6]. Most ITPNs arise in the main pancreatic duct. It is estimated that only around 5% of ITPNs are associated with branch ducts. In contrast, IPMN affects side branches in up to 86% of the cases [12]. Both tumor entities, ITPN and IPMN display a relevant malignant potential. In the series of Kolby et al., 13 out of 30 patients suffering from ITPN showed invasive tumors growth [6]. Comparing patients with invasive and with non-invasive ITPNs, they found that the median age was five years above in the group of patients with invasive ITPN. Analogous to the development in IPMNs, this observation suggests a time delay between occurrence of ITPN and malignant transformation.

Only few articles have focused on imaging findings for ITPN. Oh et al. and Ishigami et al. have emphasized upstream dilation of the main pancreatic duct due to a mass effect with subsequent obstruction in ITPN whereas IPMN typically leads to a downstream dilation of the main pancreatic duct due to hypersecretion of mucin [8]. However, in most cases preoperative diagnosis of ITPN remains difficult because it eventually resembles other tumor entities such as common ductal adenocarcinoma of the pancreas, IPMN, mucinous cystic neoplasms (MCN), acinar cell carcinoma, or tumor metastasis of renal cell carcinoma [9]. All tumors mentioned may show similar properties on imaging, particularly in case of a malignant and solid tumor growth. Motosugi et al. published the radiologic features of 11 histologically confirmed ITPNs [13]. They describe a low density of the ITPN lesion when compared to the adjacent pancreatic parenchyma in the arterial, portal venous, and delayed phase. Moreover, the distal main pancreatic duct was dilated in all patients except for one, who had a branch duct lesion. Among 49 patients with available data, the mean size of ITPN was 4.5 ± 3.3 cm (Range: 1–15 cm) [9]. At the time of diagnosis, ITPN seems to be larger and shows less frequently signs of local irresectabilty such as arterial infiltration when compared to common ductal adenocarcinoma of the pancreas of the same size [9].

In summary, it seems very difficult to identify ITPN preoperatively because no specific characteristics can be used to differentiate it reliable from other cystic or solid neoplasms of the pancreas [9]. In the present case, preoperative imaging diagnosis on the basis of CT was of a pancreatic adenocarcinoma. Unfortunately, our patient did not undergo magnetic resonance imaging (MRI) imaging before curative resection. Due to the lack of data in literature, one could only speculate that - even in case a specific MRI is available - the diagnosis of ITPN cannot be made before final histopathology of the resected specimen.

Histologically, ITPN shows a tubulopapillary growth with entirely high-grade atypical cells with no or only minimal cytoplastic mucin and no visible mucin secretion in the pancreatic ducts. In order to further distinguish ITPN from IPMN, immunohistochemistry may be helpful. ITPNs are typically positive for cytokeratin (CK) 7, CK19, MUC-1, and MUC-6, and they are negative for MUC-2, MUC-5AC, trypsin, and b-catenin. This is in contrast to IPMNs, which are usually positive for MUC-5AC but often negative for MUC-1 [14]. In contrast to IPMNs which show KRAS-Mutations in approximately 60% of cases and ductal adenocarcinoma of the pancreas which shows KRAS mutations in almost all cases [15], no KRAS mutations were observed in a series of 24 ITPNs with invasive (n = 13) or non-invasive tumor growth (n = 11). However, due to the limited number of cases available, molecular analysis still have limited significance.

Oncological surgical resection of ITPN remains the only curative management option [9]. In case of multifocal ITPN pancreatectomy has to be considered. Among 37 cases with ITPN, the overall 1-, 3-, and 5-year survival rates after surgery were 97.3, 80.7, and 80.7%, respectively. Even in case of ITPN associated carcinoma, patients revealed an excellent overall 5-year survival of 81.5%, which is more favorable when compared to ductal adenocarcinoma of the pancreas or even IPMN associated pancreatic cancer [8,9]. However, there are patients who showed local recurrent disease [16], or even died of ITPN due to multiple liver metastases [6]. The latter may indicate that oncological resection is warranted before a systemic spread of the tumor occurs.

The presented patient received adjuvant chemotherapy using gemcitabine and capecitabine (Xeloda). Since cancer arising in ITPN is a very rare tumor entity, no clinical studies are available on this topic. Kuscher et al. for example reported that in a comparable case with a pT2, pN0 (0/25), R0 tumor an interdisciplinary tumor conference decided for short time follow-up (three months) without adjuvant chemotherapy [5]. Maybe in the future, clinical studies are available to clarify this controversially discussed issue.

## 4 Conclusions

ITPN displays a rare entity of pancreatic neoplasms. As shown in the present case report, there is a relevant potential of malignant transformation and therefore radical surgical resection is warranted and displays the only curative treatment option. When compared to regular ductal adenocarcinoma, patients with ITPN reveal a lower incidence of lymph node metastasis and a better overall survival.

## Consent

Written informed consent was obtained from the patient for publication of this case report and accompanying images. A copy of the written consent is available for review by the Editor-in-Chief of this journal on request.

## Author contribution

Contributions of the authors:

Study concept and design: SF, RH, JK.

Data collection and interpretation: SF, RKS, CMS.

Writing: SF, RKS, CMS, RH, JK.

Proof reading: SF, RKS, CMS, RH, JK, KF, GR, AB.

## Guarantor

Stefan Fritz, MD accepts full responsibility for the work, conducted the study, had access to the data, and controlled the decision to publish.

## Provenance and peer review

Commissioned, externally peer-reviewed.

## Ethical approval

This is a case report without personal data or personal figures of the patient. Therefore no ethical approval is required.

## Funding

The case report was written without any funding.

## Research Registration Number

The case report does not contain data of human studies.

## Conflict of interests

The authors declare that there are no conflicts of interest.
